# A Cytochrome B_5_-Like Heme/Steroid Binding Domain Protein, PlCB5L1, Regulates Mycelial Growth, Pathogenicity and Oxidative Stress Tolerance in *Peronophythora litchii*

**DOI:** 10.3389/fpls.2021.783438

**Published:** 2021-11-25

**Authors:** Wen Li, Peng Li, Xiaofan Zhou, Junjian Situ, Yiming Lin, Jiahui Qiu, Yuling Yuan, Pinggen Xi, Zide Jiang, Guanghui Kong

**Affiliations:** ^1^Guangdong Province Key Laboratory of Microbial Signals and Disease Control, Department of Plant Pathology, South China Agricultural University, Guangzhou, China; ^2^Integrative Microbiology Research Centre, South China Agricultural University, Guangzhou, China

**Keywords:** *Peronophythora litchii*, cytochrome b5-like heme/steroid binding domain, growth, pathogenicity, oxidative stress

## Abstract

As an electron transport component, cytochrome b_5_ is an essential component of the Class II cytochrome P450 monooxygenation system and widely present in animals, plants, and fungi. However, the roles of Cyt-b_5_ domain proteins in pathogenic oomycetes remain unknown. *Peronophythora litchii* is an oomycete pathogen that causes litchi downy blight, the most destructive disease of litchi. In this study, we identified a gene, designated *PlCB5L1*, that encodes a Cyt-b_5_ domain protein in *P*. *litchii*, and characterized its function. *PlCB5L1* is highly expressed in the zoospores, cysts, germinated cysts, and during early stages of infection. *PlCB5L1* knockout mutants showed reduced growth rate and β-sitosterol utilization. Importantly, we also found that *PlCB5L1* is required for the full pathogenicity of *P*. *litchii*. Compared with the wild-type strain, the *PlCB5L1* mutants exhibited significantly higher tolerance to SDS and sorbitol, but impaired tolerance to cell wall stress, osmotic stress, and oxidative stress. Further, the expression of genes involved in oxidative stress tolerance, including peroxidase, cytochrome P450, and laccase genes, were down-regulated in *PlCB5L1* mutants under oxidative stress. This is the first report that a Cyt-b_5_ domain protein contributes to the development, stress response, and pathogenicity in plant pathogenic oomycetes.

## Introduction

Cytochrome b_5_-like heme/steroid binding domain (Cyt-b_5_ domain) proteins have been found in all major eukaryotic lineages and a few bacteria (Mifsud and Bateman, 2002). As a physiological electron transport component, cytochrome b_5_ is an essential component of the Class II cytochrome P450 monooxygenation system (Hlavica, 1984; Zhang and Scott, 1994; Porter, 2002). Cytochrome b_5_ also interacts with various proteins, including cytochrome b_5_ reductase, sucrose or sorbitol transporters, cytochrome c (Davydov, 2001; Schenkman and Jansson, 2003; Fan et al., 2009; Gentry et al., 2019) in multiple biological reactions, participating in fatty acid desaturation (Takashi et al., 1972), fatty acid prolongation (Keyes et al., 1979), apoptosis and catabolism of exogenous organisms and drugs (Kandel and Lampe, 2014). Some cytochrome b_5_ fuse with additional domains, such as fatty acid desaturase, sulfite oxidase, nitrate reductase, or chitin synthase (Sayanova et al., 1997; Rudolph et al., 2003; Ikeyama et al., 2010; Kong et al., 2012).

In *Arabidopsis thaliana*, a Cyt-b_5_ domain protein, RLF, is involved in lateral root initiation, while RLF and its homologs are highly conserved among a variety of plant species (Ikeyama et al., 2010). In vertebrates, progesterone receptor membrane component 1 (PGRMC1) is a membrane-bound progesterone receptor containing a Cyt-b_5_ domain, relating to steroid synthesis and metabolism (Mifsud and Bateman, 2002; Kimura et al., 2012). Furthermore, Cytochrome P450s and the cytochrome b_5_ reductase-cytochrome b_5_ system have been reported to play an important role in the synthesis of sterols in fungi, which are indispensable for fungal hyphal growth and asexual sporulation (Lamb et al., 1999; Derbyshire et al., 2015). In *Saccharomyces cerevisiae*, heterologous expression of *Aspergillus oryzae* D9D genes, *AoD9D1* and *AoD9D2*, which encode proteins containing fatty acid desaturase and Cyt-b_5_ domain, contribute to the accumulation of unsaturated fatty acid and tolerance of high salinity stress depending on both the fatty acid desaturase and Cyt-b_5_ domain (Li et al., 2019). However, our knowledge of the function of Cyt-b_5_ domain proteins in plant pathogenic oomycetes is still lacking.

Oomycetes include many notorious plant and animal pathogens, resulting in significant global losses in agriculture, forestry, and aquaculture (Thines, 2018). Litchi downy blight caused by the oomycete pathogen, *Peronophythora litchii*, is a major disease of litchi and leads to huge production losses (Jiang et al., 2017; Liu et al., 2017; Kong et al., 2020). The *P. litchii* infects fruits, flowers, leaves, and twigs, causing panicle rot, fruit brown, and leaf blight (Kong et al., 2021). The recent publication of *P. litchii* genome has greatly accelerated the molecular research of this pathogen (Ye et al., 2016). *P. litchii* PlAvh142, PlPAE5, PlBZP32, PlM90, PlMAPK2, and PlMAPK10 were found to be associated with the development and/or virulence of *P. litchii* (Jiang et al., 2018; Kong et al., 2019, 2020; Situ et al., 2020a; Huang et al., 2021). However, the molecular mechanisms of development and pathogenicity of *P*. *litchii* are still largely unknown.

A better understanding of the development and virulence of *P*. *litchii* is crucial for the control of litchi downy blight. In this study, we identified a Cyt-b_5_ domain protein, PlCB5L1, in *P*. *litchii* and carried out functional characterization of PlCB5L1. We found that *PlCB5L1* is conserved in oomycetes and up-regulated in zoospores, cysts, germinated cysts, and early stages of infection. We knocked out this gene by CRISPR/Cas9 technique and found that PlCB5L1 contributed to mycelial growth and utilization of β-sitosterol. Meanwhile, deletion of PlCB5L1 impaired the H_2_O_2_ tolerance, laccase activity and pathogenicity of *P*. *litchii*. Further study showed that expression of peroxidase, cytochrome P450, and laccase genes were down-regulated in *PlCB5L1* knockout mutants. The study demonstrated the role of PlCB5L1 in growth, H_2_O_2_ tolerance, and pathogenicity.

## Materials and Methods

### Identification and Phylogenetic Analysis of Cyt-b_5_ Domain Proteins

The genome sequence and gene annotations of *P. litchii* were obtained from NCBI (BioProject ID: PRJNA290406). Translated protein sequences of all *P. litchii* genes were analyzed using InterProScan5 (version 5.46) to identify proteins with the cytochrome b_5_-like heme/steroid binding domain. The amino acid sequence alignment was generated and adjusted in BioEdit (version 7.0.9.1).

### *Peronophythora litchii* Strain and Culture Conditions

*P*. *litchii* wild type (WT) strain SHS3 (Ye et al., 2016), the CK strain, and Δ*plcb5l1* mutants were cultured on carrot juice agar (CJA) medium (juice from 300 g carrot for 1 L medium, 15 g agar/L for solid media) at 25°C in darkness. The control (CK) strain is a transformant that failed to knockout *PlCB5L1*. Litchi leaves were harvested from healthy litchi trees in an orchard in South China Agricultural University, Guangzhou, Guangdong province, China. For sporangia production, five 9 mm diameter mycelial plugs were flushed with 2 mL sterilized water, filtering the subsequent suspension with a 100 μm mesh filter. The suspension was incubated at 16°C for 1 h, for zoospores release. After shaking the suspension for 30 s on a vortex oscillator, zoospores were encysted. Cysts were incubated at 25°C 60 rpm for 0.5 h, for cysts germinating. The number of sporangia, release rate of zoospores, and germination rate of cysts were counted under a microscope. The number of oospores was calculated from three 9 mm diameter zones, at 10th days after inoculating on CJA medium at 25°C in the dark (Jiang et al., 2017).

### Nucleic Acid Extraction and Quantitative Reverse Transcription PCR

Fungal genomic DNA was extracted from mycelia grown in CJA medium or infected litchi leaves using a Fungal DNA Kit (Omega, America). PCR amplification was performed using Phanta Max Super-Fidelity DNA Polymerase (Vazyme, China). PCR product purification was performed using Cycle Pure Kit (Omega, America) or Gel Extraction Kit (Omega, America). Total RNAs from the life cycle stages of *P. litchii*, including mycelia, sporangia, zoospores, cysts, germinated cysts, oospores, and stages of infection, were extracted using All-In-One DNA/RNA Mini-preps Kit (Bio Basic, China). FastKing RT Kit (TIANGEN, China) was used for the first-strand cDNA synthesis. The cDNA was stored at –20°C. The expression profile of *PlCB5L1* was analyzed with qRT-PCR using SYBR^®^ Premix Ex Taq™ II (TaKaRa, Japan) and primers *PlCB5L1*-qRTF/R. *P. litchii* actin gene (*PlActin*) (Jiang et al., 2017) was used as a loading control and the relative fold change was calculated using the 2^–ΔΔCT^ method (Livak and Schmittgen, 2001). Primers used for these analyses were listed in Supplementary Table 1.

### CRISPR/Cas9 Editing for *PlCB5L1* Knockout

**FIGURE 1:**
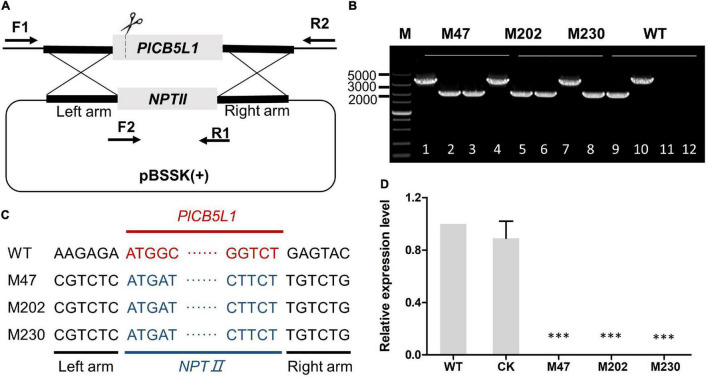
Gene knockout of *PlCB5L1*. **(A)** CRISPR/Cas9-mediated knockout of *PlCB5L1*, in which the *NPTII* gene replaces the *PlCB5L1.* The primers (F1, R1, and F2, R2) used for PCR analysis are indicated by horizontal arrows. F1: Left-out-F; F2: NPTII-F; R1: NPTII-R; R2: Left-out-R (Supplementary Table 1). **(B)** Genomic DNA PCR analysis of the Δ*plcb5l1* mutants. Marker: DL 5000. Lane 1, lane 4, lane 7, and lane 10 were amplifying with primers F1/R2. Lane 2, lane 5, lane 8, and lane 11 were amplifying with primers F1/R1. Lane 3, lane 6, lane 9, and lane 12 were amplifying with primers F2/R2. **(C)** Sequence analysis of the Δ*plcb5l1* mutants. **(D)** Relative gene expression levels of *PlCB5L1* in mutants and CK strains. The expression level of WT was set as 1. Data are mean ± SD (*n* = 9). Asterisks represent significant difference (***p < 0.001) based on statistics analysis using Student’s *t*-test.

A SgRNA was selected and inserted into the sgRNA vector pYF2.3G-RibosgRNA as previously described (Fang and Tyler, 2016; Situ et al., 2020b). To generate gene replacement constructs, 1 kb long upstream/downstream arms of the *PlCB5L1* coding region were inserted into pBluescript II KS vector using the ClonExpress MultiS One Step Cloning Kit (Vazyme, China) (Figure 1A). The pYF2.3G-RibosgRNA (*PlCB5L1*) vector, the hSPCas9 vector pYF2-PsNLS-hSpCas9, and the pBluescript II KS (*PlCB5L1*) vector were co-transformed into protoplasts of strain SHS3 using PEG-mediated protoplast transformation (Fang and Tyler, 2016). Preliminary transformants were screened by CJA medium containing 50 μg/mL G418. These transformants were further verified by genomic PCR and sequencing. These primers were listed in Supplementary Table 1.

### Pathogenicity Assays on Litchi Leaves

Pathogenicity assays were performed by inoculating 10 μL (20 zoospores per μL) of zoospore suspensions of *P. litchii* WT, CK, and Δ*plcb5l1 mutants* (M47, M202 and M230) on the abaxial side of litchi leaves at 25°C in the dark. Lesion diameters were measured and photographed 48 h after inoculation. The relative biomass was determined by the ratio of *P. litchii* DNA to litchi DNA in the inoculated tissues by qRT-PCR using the specific primers for *P. litchii* and litchi *Actin* genes (Supplementary Table 1; Zhong et al., 2011). The significant differences were analyzed with Student’s *t*-test and three independent replicates were set up, with at least 6 leaves in each replicate.

### Sensitivity to Various Stress

To investigate the sensitivity of *PlCB5L1* mutants under different stress conditions, the mycelial plugs (diameter = 9 mm) of Δ*plcb5l1* mutants were inoculated in the center of the Plich medium (van West et al., 1999) and cultured at 25°C in the dark for 7 days. The Plich media were supplemented with different concentrations of sodium dodecyl sulfate (SDS), Congo Red (CR), Calcofluor White (CFW), H_2_O_2_, sorbitol, NaCl or CaCl_2_. WT and CK strains were used as control. The growth inhibition rate was calculated as: growth inhibition rate (%) = (growth diameter on stress-free plates—growth diameter on stress plates)/growth diameter on stress-free plates × 100%.

To analyze the expression of *PlCB5L1* under oxidative stress, the WT strain was cultured in liquid Plich medium for 3 days at 25°C in the dark. The mycelia were immersed in the liquid medium supplemented with 5 mM H_2_O_2_ for 0, 5, 15 or 55 min. All samples were harvested and the expressional levels of *PlCB5L1* were evaluated by qRT-PCR.

### Diaminobenzidine Staining

DAB staining was performed to visualize the accumulation of reactive oxygen species (ROS) in the infected leaves. The infected leaves were stained with 1 mg/mL DAB solution at room temperature in the dark for 8 h, and then decolorized in 96% ethanol for 48 h (Molina and Kahmann, 2007). ImageJ was used to record the grayscale values of all pixels within the brown areas in the infected leaves.

## Results

### Phylogenetic Analysis and the Transcriptional Profiles of *PlCB5L1*

**FIGURE 2:**
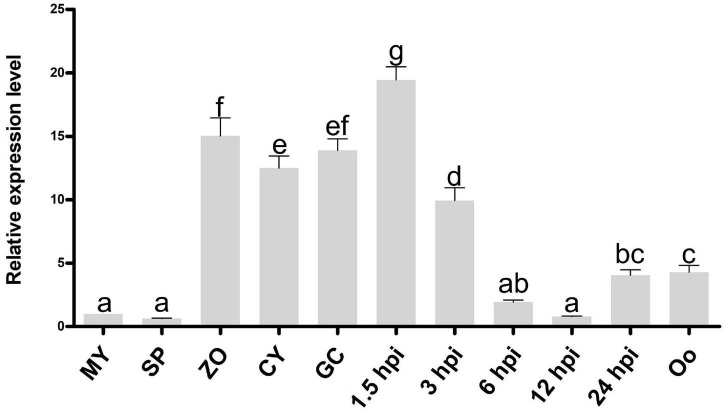
Expression pattern of *PlCB5L1* during the sexual, asexual and infection stages of *P*. *litchii*. Expression levels were determined by qRT-PCR using RNAs extracted from vegetative mycelia (MY), sporangia (SP), zoospores (ZO), cysts (CY), germinated cysts (GC), oospores (Oo), and samples from 1.5, 3, 6, 12, and 24 h post-inoculation with zoospores on leaves. The relative expression levels were calculated by using the MY sample as reference. Data are mean ± SD (*n* = 9). The data were statistically analyzed with SPSS (version 20.0) with Duncan’s Multiple Range Test method and different letters denote statistical differences (*p* < 0.05).

We screened all proteins encoded in the genome of *P. litchii* (Ye et al., 2016) using InterProScan and identified 11 Cyt-b_5_ superfamily members. In addition to the cytochrome b_5_-like heme/steroid binding domain (Cyt-b_5_), some proteins also contain additional domains, including the flavin adenine dinucleotide domain (FAD), fatty acid desaturases domain (FA_desaturase), molybdopterin cofactor oxidoreductase dimerization domain (Mo-co_dimer), cGMP-specific phosphodiesterases, adenylyl cyclases, and FhlA (GAF) domain, and oxidoreductase molybdopterin binding domain (Oxidored_molyb) domain (Supplementary Figure 1). Among them, *Pl109805* (named *PlCB5L1*) showed the highest transcriptional levels during infection among the 11 Cyt-b_5_ superfamily members, and is dramatically up-regulated in the stages of infection (based on unpublished transcriptome data). Furthermore, the expression profile of the *PlCB5L1* were determined by quantitative reverse-transcription polymerase chain reaction (qRT-PCR). Compared with mycelial stage, *PlCB5L1* had much higher expression levels in zoospores, cysts, cyst germination, and the early stages of plant infection (1.5 and 3 h post-inoculation, hpi) (Figure 2), suggesting that PlCB5L1 may have important roles in the colonization and early infection of *P. litchii*. Therefore, we characterized the function of PlCB5L1 in this study.

**FIGURE 3:**
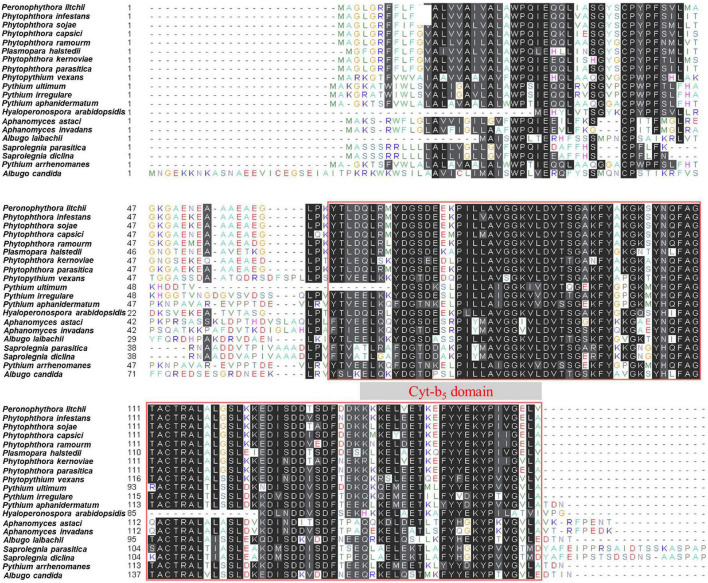
Domain arrangement of PlCB5L1 protein and its orthologs. Protein sequence alignment of PlCB5L1 and its orthologs from 19 oomycete species. Columns with identical and similar amino acid sequences were colored black and gray, respectively. The red box indicates Cyt-b_5_ domain.

*PlCB5L1* encodes a protein of 160 amino acids (aa) and its Cyt-b_5_ domain is located in the C-terminal of the protein (64–160 aa). Sequence analyses showed that a single ortholog of PlCB5L1 is present in each of the 19 sequenced oomycetes and the levels of protein sequence similarity between PlCBL5L1 and its orthologs range between 51.70 and 96.30%, indicating that PlCB5L1 is well-conserved in oomycetes (Supplementary Table 2 and Figure 3). We also compared PlCB5L1 with its most similar homologs in other eukaryotes including fungi, animals, and plants, they possess high levels of sequence divergence (protein sequence similarity < 45%) (Supplementary Table 2).

### Generation of *PlCB5L1* Knockout Mutants by CRISPR/Cas9 Genome Editing Method

We generated three *PlCB5L1* knockout mutants (M47, M202, and M230) using the CRISPR/Cas9 system, as previously described (Fang and Tyler, 2016; Situ et al., 2020b; Figure 1A). Genomic PCR assays and sequencing results proved that *PlCB5L1* was replaced with the *NPTII* gene in the three mutants (Figures 1B,C). Subsequently, qRT-PCR analysis confirmed that *PlCB5L1* was not expressed in these mutants (Figure 1D). A transformant that failed to knockout *PlCB5L1* was selected as the control (CK) strain.

### *PlCB5L1* Is Required for the Growth and Utilization of β-Sitosterol

**FIGURE 4:**
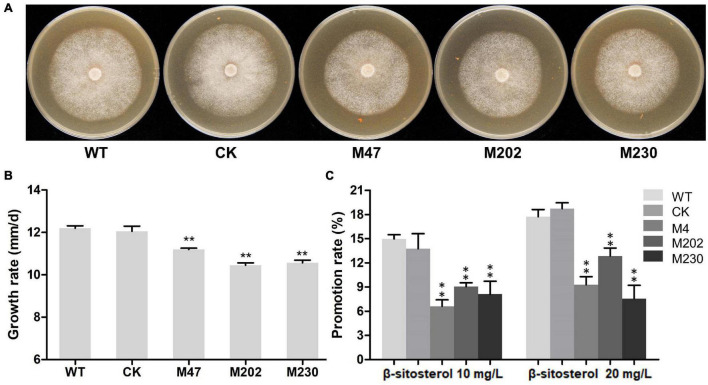
Growth rate and β-sitosterol utilization of WT, CK, and the Δ*plcb5l1* mutants. **(A)** Colonies of three independent Δ*plcb5l1* mutants (M47, M202, and M230), WT and CK strains were cultured on CJA medium at 25°C in the dark for 5 days. **(B)** Growth-rates were measured on CJA medium. **(C)** Promotion ratio (%) of WT, CK, and Δ*plcb5l1* mutants under the condition of Plich medium adding 10 mg/L or 20 mg/L β-sitosterol. The values are mean ± SD (*n* = 9). The data were statistically analyzed with Duncan’s Multiple Range Test method, with significant differences defined as **p < 0.01.

To investigate the biological functions of *PlCB5L1*, the sexual and asexual phenotypes of the Δ*plcb5l1* mutants, CK and wild-type strains (WT) were examined. We cultured the Δ*plcb5l1* mutants, WT and CK strains on CJA medium for 5 days at 25°C in the dark, and measured the colony diameter of each strain. As shown in Figures 4A,B, the average growth rates (mm/day) of the mutants (10.45–11.20) were significantly lower than that of the WT (12.19) and CK (12.05), suggesting that *PlCB5L1* contributes to the vegetative growth of *P. litchii*.

Previous studies have shown that *Phytophthora* spp. cannot synthesize sterols on their own; instead, they use sterol carrier protein elicitins to absorb and metabolize many kinds of sterols from host plants (Boissy et al., 1999; Dahlin et al., 2017). β-sitosterol is one of the most abundant sterols within plant tissues and is useful for mycelial growth of *P. infestans* in V8 agar medium (Medina and Platt, 1999; Klingberg et al., 2008; Stong et al., 2013). We examined the function of *PlCB5L1* on β-sitosterol utilization by culturing Δ*plcb5l1* mutants, WT, and CK on Plich medium supplemented with 10 mg/L or 20 mg/L β-sitosterol. Their colony diameters were measured 5 days after inoculation at 25°C in the dark. Results showed that the growth-promoting rates of WT and CK were significantly (*p* < 0.05) higher than that of mutants on Plich medium with 10 or 20 mg/L β-sitosterol (Figure 4C), suggesting that the Δ*plcb5l1* mutants have impaired capability of β-sitosterol utilization compared with WT and CK.

Next, we evaluated the sporangia number, sporangia size, the rate of zoospores release and cysts germination, as well as the number of produced oospores. Our results showed that the knockout of *PlCB5L1* did not have a significant impact on these phenotypes (Supplementary Figure 2).

### *PlCB5L1* Is Required for the Full Virulence of *P. litchii*

**FIGURE 5:**
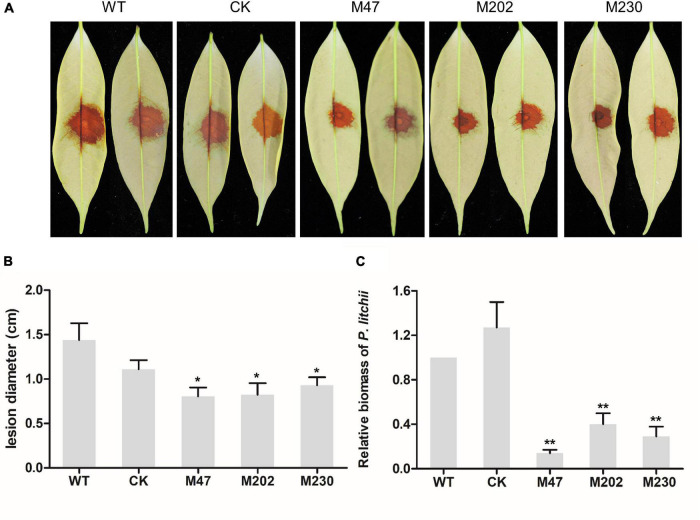
Pathogenicity test of *P. litchii* Δ*plcb5l1* mutants, WT and CK. **(A)** Litchi leaves were inoculated with 200 zoospores of WT, CK, three Δ*plcb5l1* mutants M47, M202, and M230 for 48 h at 25°C in the dark. Images showed representative leaves for each instance. **(B)** Lesion lengths were measured 2 dpi. Data are mean ± SD (*n* = 9) (*p < 0.05; Duncan’s multiple range test). **(C)** Relative *P. litchii* biomass was measured to evaluate the severity of infection by qRT-PCR. Data are mean ± SD (*n* = 9). Asterisks represent significant difference (**p < 0.01; *t*-test range test).

To determine the role of *PlCB5L1* in the pathogenicity of *P. litchii*, the abaxial surface of tender litchi leaves were inoculated with zoospores suspensions (20 per μL) of WT, CK, and Δ*plcb5l1* mutants (M47, M202, and M230), and kept at 25°C. At 2 days post-inoculation (dpi), we measured the lesion diameter (Figure 5A) and found that the lesions caused by Δ*plcb5l1* mutants were significantly (*p* < 0.05) smaller than WT and CK strains (Figure 5B). Biomass quantification confirmed that the amounts of Δ*plcb5l1* mutants DNA in litchi leaves were at least 40% lower than that of WT and CK (Figure 5C). These results suggest that *PlCB5L1* is required for the full virulence of *P. litchii*.

### *PlCB5L1* Is Involved in Cell Wall Integrity and Tolerance to H_2_O_2_, Osmotic and Salt Stress

**FIGURE 6:**
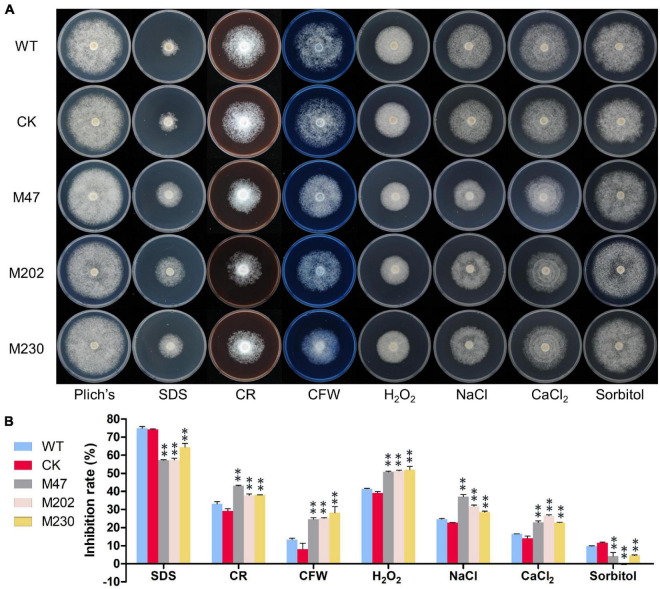
*PlCB5L1* is involved in stress tolerance. **(A)** Assay of mycelial growth of WT, CK, and Δ*plcb5l1* mutants on Plich medium only or supplemented with 25 μg/mL SDS, 350 μg/mL CR, 100 μg/mL CFW, 2.0 mM H_2_O_2_, 0.05 M NaCl, 0.1 M CaCl_2_, and 0.2 M Sorbitol. Images were taken 7 days post inoculation. **(B)** Colony diameters were measured 7 days after inoculation. Rates of growth inhibition were calculated for each treatment relative to growth rate on Plich medium. Data are mean ± SD (*n* = 9). and asterisks denote significant differences between Δ*plcb5l1* mutants and the WT (**p < 0.01; Duncan’s multiple range test).

To investigate whether *PlCB5L1* is related to various abiotic stress responses of *P. litchii*, the Δ*plcb5l1* mutants, WT and CK strains were cultured on Plich medium supplemented with different concentrations of sodium dodecyl sulfate (SDS), Congo red (CR), calcofluor white (CFW), H_2_O_2_, sorbitol, NaCl or CaCl_2_. Colony diameter was measured after 7 days of growth at 25°C in the dark (Figure 6A).

The growth inhibition rates of Δ*plcb5l1* mutants were significantly (*p* < 0.01) lower than that of WT and CK under the cell wall stress caused by 25 μg/mL SDS and the osmotic stresses caused by 0.2 M sorbitol (Figures 6A,B). On the other hand, Δ*plcb5l1* mutants were more sensitive to the cell wall stress caused by 350 μg/mL CR and 100 μg/mL CFW, the oxidative stress caused by 2.0 mM H_2_O_2_, and the salt stress caused by 0.05 M NaCl and 0.1 M CaCl_2_ (Figures 6A,B). These results suggest that the function of *PlCB5L1* is related to osmoregulation, cell wall integrity, and tolerance to salt and H_2_O_2_.

### *PlCB5L1* Is Required for Detoxifying the Plant Oxidative Burst

**FIGURE 7:**
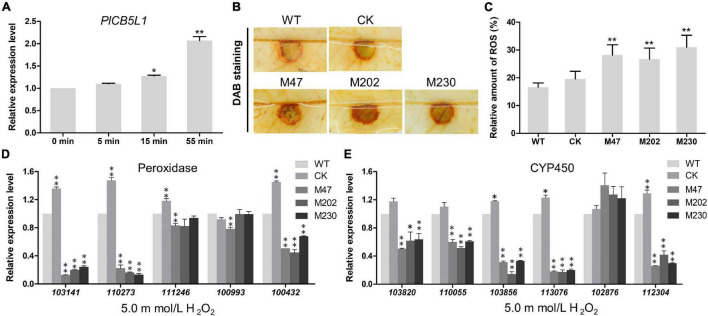
*PlCB5L1* is associated with the accumulation of ROS and the expression of peroxidase- and cytochrome P450-encoding genes. **(A)** Transcriptional analysis of the *PlCB5L1* gene during oxidative stress (5 mM H_2_O_2_, for 0, 5, 15, 55 min). Expression levels were normalized using the values at 0 min as “1.” Asterisks represent significant difference, and Data are mean ± SD (*n* = 9). **(B)** ROS accumulation of litchi leaves was detected by DAB staining at 18 h after inoculation. **(C)** The DAB staining was quantified by ImageJ, which was used to record the grayscale values of all pixels within the brown areas. Data are mean ± SD (*n* = 9). **(D,E)** qRT-PCR analysis of *P. litchii* putative peroxidase-encoding genes and cytochrome P450-encoding genes in Δ*plcb5l1* mutants, CK and WT strain under oxidative stress conditions (5 mM H_2_O_2_, for 5 min). Data are mean ± SD (*n* = 9). Asterisks represent significant differences (*p < 0.05 or **p < 0.01) based on Student’s *t*-test.

Our abovementioned results showed that Δ*plcb5l1* mutants were more sensitive to H_2_O_2_ than WT and CK (Figures 6A,B). To investigate the expression levels of *PlCB5L1* under oxidative stress, the hyphae of WT were exposed to 5 mM H_2_O_2_-added Plich medium for 0, 5, 15, and 55 min, in order to simulate oxidative stress imposed by the host upon infection. The qRT-PCR analysis showed that *PlCB5L1* expression is significantly up-regulated (*p* < 0.05) at 15 and 55 min after H_2_O_2_ treatment (Figure 7A).

Since ROS are known to play a key role in many plant-pathogen interactions (Lamb and Dixon, 1997), ROS accumulation was detected by DAB staining. There is a higher level of H_2_O_2_ accumulation in litchi leaves inoculated with Δ*plcb5l1* mutants compared with WT and CK strains at 18 h post-inoculation (Figures 7B,C), suggesting that the Δ*plcb5l1* mutants showed lower capability of scavenging ROS.

### Knockout of *PlCB5L1* Attenuates the Expression Levels of Extracellular Peroxidases and Cytochrome P450 Genes

To further investigate the role of *PlCB5L1* in scavenging host-derived ROS, we examined the expression levels of five peroxidases and six cytochrome P450 (CYP) genes in WT strain and the Δ*plcb5l1* mutants. These genes possess highly expression levels in WT strain, based on RNA-seq data (Unpublished). After soaking mycelia in 5 mM H_2_O_2_-added Plich medium for 5 min, the expression levels of three peroxidase genes (*Pl101341*, *Pl110273*, *Pl100432*) (Figure 7D) and five CYP genes (*Pl113076*, *Pl110055, Pl103820*, *Pl103856*, *Pl112304*) were significantly (32∼83 and 36∼86%, respectively) lower in Δ*plcb5l1* mutants comparing with WT (Figure 7E). These results suggest that *PlCB5L1* can affect the expression of these peroxidase genes and CYP genes under oxidative stress.

### Knockout of *PlCB5L1* Can Weaken the Activity of Extracellular Laccase and Decrease the Expression of Laccase-Encoding Gene

**FIGURE 8:**
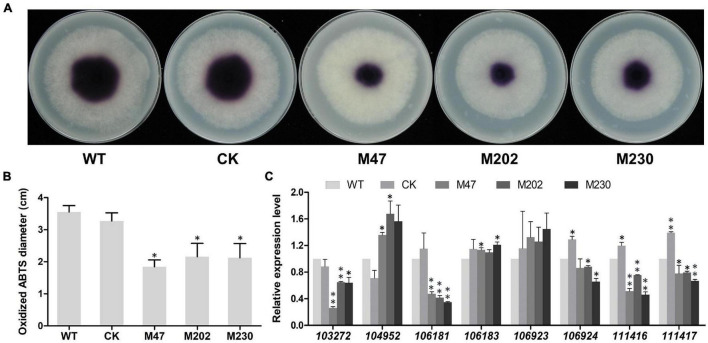
Knockout of *PlCB5L1* decreased the activity of extracellular laccase and transcription levels of laccase-encoding genes. **(A)** Laccase activity was determined by monitoring oxidized ABTS (purple color) in LBA media supplemented with 0.2 mM ABTS, photographs were taken 7 days after inoculation. **(B)** The diameters of oxidized ABTS (dark purple) were measured at 7 days post inoculation. **(C)** Relative expression of eight laccase-encoding genes in WT (set at 1), CK and Δ*plcb5l1* mutants under oxidative stress conditions, 5 mM H_2_O_2_ for 5 min. Data are mean ± SD (*n* = 9, **p < 0.01, *p < 0.05; Student’s *t*-test).

Extracellular laccase activity has been shown to be an important component of plant pathogens defense against oxidative stress (Mayer and Staples, 2002; Yang et al., 2012), therefore we analyzed the laccase activity in Δ*plcb5l1* mutants, WT and CK strains following an established protocol (Sheng et al., 2015). In the 0.2 mM 2,2-azobis (3-ethylbenzothiazoline-6-sulfonic acid) (ABTS)-added lima bean agar (LBA) medium, the three mutants accumulated significantly (*p* < 0.05) lower amounts of oxidized ABTS than WT and CK strains, at 7 days after inoculation, suggesting that Δ*plcb5l1* mutants had lower laccase activity (Figures 8A,B).

We then examined whether this reduction in laccase activity was due to the down-regulation of laccase genes. We focused on eight laccase genes (*Pl103272*, *Pl104952*, *Pl106181, Pl106183, Pl106923*, *Pl106924*, *Pl111416*, and *Pl111417*), which were selected because they are highly expressed in WT during oxidative stress (5 mM H_2_O_2_) and their proteins possess signal peptides (Huang et al., 2021). In Δ*plcb5l1* mutants, the expression levels of four (*Pl103272*, *Pl106181*, *Pl111416*, and *Pl111417*) laccase-encoding genes were significantly decreased (Figure 8C).

## Discussion

Cytochrome b_5_-like heme/steroid binding domain proteins are widely present in eukaryotes. The interactions between Cyt-b_5_ domain proteins and various proteins, such as progesterone receptor membrane component 1 and 2, chitin synthases, fatty acid desaturases, have important roles in multiple biological processes (Napier et al., 1999; Mifsud and Bateman, 2002; Kimura et al., 2012; Kong et al., 2012). In this study, we identified a member of the *P. litchii* cytochrome b_5_ superfamily, *PlCB5L1*, that is well conserved in all sequenced oomycetes. Knockout of *PlCB5L1* resulted in reduced mycelial growth rate and utilization of β-sitosterol, significantly attenuated *P. litchii* virulence on litchi plants, as well as substantially altered expression of peroxidase, cytochrome P450, and laccase genes. This is the first report that a Cyt-b_5_ domain protein contributes to growth, β-sitosterol utilization, virulence, and oxidative response in plant pathogenic oomycetes.

Some Cyt-b_5_ domain proteins (e.g., PGRMC1 and ZtCytb_5_) are involved in sterol biosynthesis and ZtCytb_5_ is necessary to the mycelial growth in *Zymoseptoria tritici* (Kimura et al., 2012; Derbyshire et al., 2015). Here, we found that the mycelial growth rates of the Δ*plcb5l1* mutants were significantly lower than that of WT (Figures 4A,B), and the mutants also showed impaired utilization of β-sitosterol (Figure 4C). We infer that the reduction in growth rate may be caused by the decrease of lipid rafts, which are highly enriched in sterols and sphingolipids (Martin and Konopka, 2004). Cytochrome b_5_ reductase—cytochrome b_5_ system plays a major role in the electron transfer of desaturase and hydroxylase; it can catalyze the biosynthesis of sphingomyelin, sterols, and various unsaturated fatty acid (Michaelson et al., 2013). However, PlCB5L1 protein has a unique Cyt-b_5_ structure and does not contain a distinct domain with potential enzymatic activity, suggesting that PlCB5L1 may not participate in the biosynthesis of sterols. *Phytophthora* spp. cannot synthesize sterols on its own, but can instead absorb and metabolize many kinds of sterols from host plants by sterol carrier protein elicitins, which are associated with mycelial growth (Nes and Stafford, 1983; Boissy et al., 1999; Gaulin et al., 2010; Dahlin et al., 2017). Therefore, we inferred that PlCBL1 might be a component for the utilization of sterols.

Under high concentrations of salt, which disrupt cell homeostasis and cause osmotic stress, microorganisms would increase the levels of unsaturated fatty acids to preserve normal membrane fluidity (Srivastava et al., 2014). Cytochrome b_5_ proteins have been found to be involved in fatty acid desaturation (Takashi et al., 1972). For instance, Li et al. (2019) transformed Cyt-b_5_ domain of the delta-9 fatty acid desaturase from *A. oryzae* into *S. cerevisiae* and the transformed strains showed enhanced accumulation of unsaturated fatty acid and stronger salt tolerance than the wild type. Similarly, our results here showed that Δ*plcb5l1* mutants were more sensitive to the salt stress caused by NaCl and CaCl_2_ (Figure 6), which might be caused by the disruption of fatty acid desaturation in the Δ*plcb5l1* mutants.

In host plants, one characteristic early defense response is the rapid production and accumulation of peroxides after the perception of non-toxic signals of pathogens (Lamb and Dixon, 1997). On the other hand, pathogens have also evolved mechanisms to scavenge host ROS by producing peroxidases, cytochrome P450s, and laccases (Molina and Kahmann, 2007; Chi et al., 2009; Matteis et al., 2012; Yang et al., 2012). In our study, we found that the expression level of *PlCB5L1* was significantly up-regulated after 15 min of H_2_O_2_ treatment (Figure 7A), suggesting that *PlCB5L1* might be involved in the oxidative stress response of *P. litchii*. Additionally, the expression levels of three peroxidase genes and five cytochrome P450 genes were significantly lower in the *PlCB5L1* knockout mutants than that of WT strain, under oxidative stress conditions (Figures 7D,E). The function of cytochrome b_5_-cytochrome b_5_ reductase electron transfer system in cytochrome P450 monooxygenation has long been recognized in animals and fungi (Syed et al., 2011). There is also a report that Cytb_5_ electron transfer plays a role in peroxidase activity (Bidlack, 1980). We infer that *PlCB5L1* is related to the expression of these peroxidase genes and cytochrome P450 genes during oxidative stress.

We also found that one CYP-encoding gene (*Pl112304*) which showed significantly decreased expression in Δ*plcb5l1* mutants is a homolog of pisatin demethylase (GenBank ID: AAR32716.1). Pisatin demethylase (PDA), the enzyme responsible for detoxifying pisatin, is a substrate-inducible CYP-encoding gene (George et al., 1998; Coleman et al., 2011). It is therefore possible that the decrease of virulence of Δ*plcb5l1* mutants is related to the decrease of degradation ability of antimicrobial compounds. However, the detailed mechanism needs to be further verified.

Overall, our study represents the first report that cytochrome b_5_ superfamily member PlCB5L1 is associated with growth and β-sitosterol utilization. PlCB5L1 also plays a key role in pathogenesis and response to oxidative stress, likely *via* the affecting activity of laccase and the expression of genes encoding peroxidases, cytochrome P450s, and laccases. The functional characterization of *PlCB5L1* provides new insights into the mycelial growth, stress resistance, pathogenesis, and laccase activity of *P. litchii*. Future studies are needed to elucidate the roles of PlCB5L1 in the growth, development, and pathogenicity of *P. litchii via* functionally interacting with P450, laccase, and peroxidase.

## Data Availability Statement

The original contributions presented in the study are included in the article/Supplementary Material, further inquiries can be directed to the corresponding author/s.

## Author Contributions

ZJ, GK, WL, and XZ designed experiments. WL, PL, XZ, JS, YL, YY, and JQ performed the experiments. WL and GK analyzed the data. GK, ZJ, and WL discussed and developed the study. WL, XZ, GK, and ZJ wrote the manuscript. All authors contributed to the article and approved the submitted version.

## Conflict of Interest

The authors declare that the research was conducted in the absence of any commercial or financial relationships that could be construed as a potential conflict of interest.

## Publisher’s Note

All claims expressed in this article are solely those of the authors and do not necessarily represent those of their affiliated organizations, or those of the publisher, the editors and the reviewers. Any product that may be evaluated in this article, or claim that may be made by its manufacturer, is not guaranteed or endorsed by the publisher.
