# Supplementary material for: A Cytochrome B5-Like Heme/Steroid Binding Domain Protein, PlCB5L1, Regulates Mycelial Growth, Pathogenicity and Oxidative Stress Tolerance in Peronophythora litchii
Source: Front Plant Sci. 2021 Nov 25;12:783438. doi: 10.3389/fpls.2021.783438 (PMC8655872; doi:10.3389/fpls.2021.783438)
Supplement: Supplementary file 1 [file Data_Sheet_1.docx]

Supplementary Material

Supplementary Table S1 The primers used in this study (see separate file)

Supplementary Table S2 PlCB5L1 orthologous proteins in oomycetes, fungal, animal and plant (see separate file)


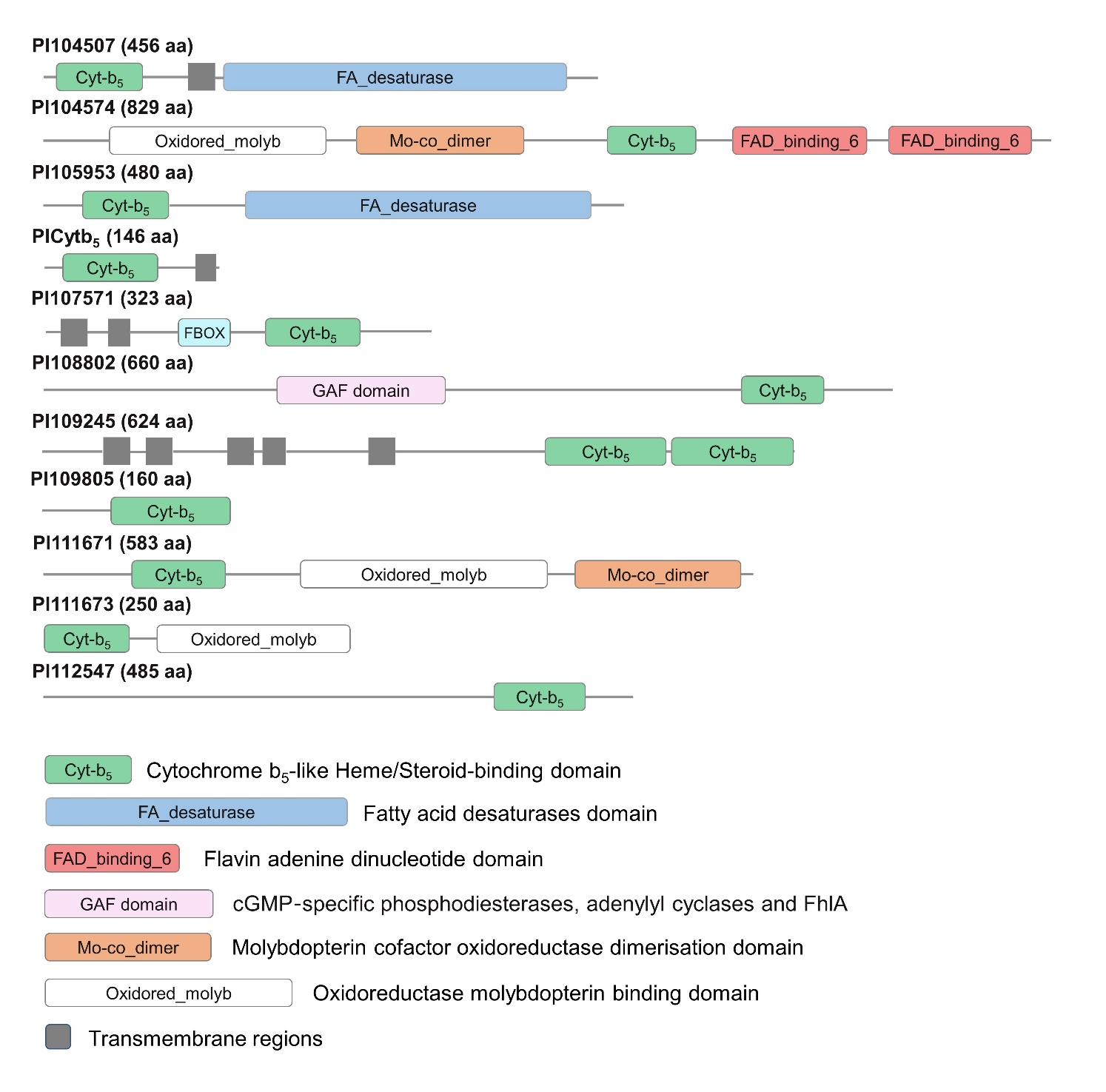


Supplementary Figure S1. A schematic figure showing the architectures of proteins containing Cyt-b_5_ domains in *P*. *litchii*. All domain names correspond to homonymous SMART database and were annotated. Cytochrome b_5_-like Heme/Steroid-binding domain (Cyt-b_5_) is indicated by a green box, and gray rectangles represent transmembrane regions. FA_desaturase (fatty acid desaturases domain), FAD_binding 6 (flavin adenine dinucleotide domain), GAF domain (cGMP‐specific phosphodiesterases, adenylyl cyclases and FhlA), Mo-co_dimer (molybdopterin cofactor oxidoreductase dimerisation domain), Oxidored_molyb (oxidoreductase molybdopterin binding domain) were also indicated in this figure.


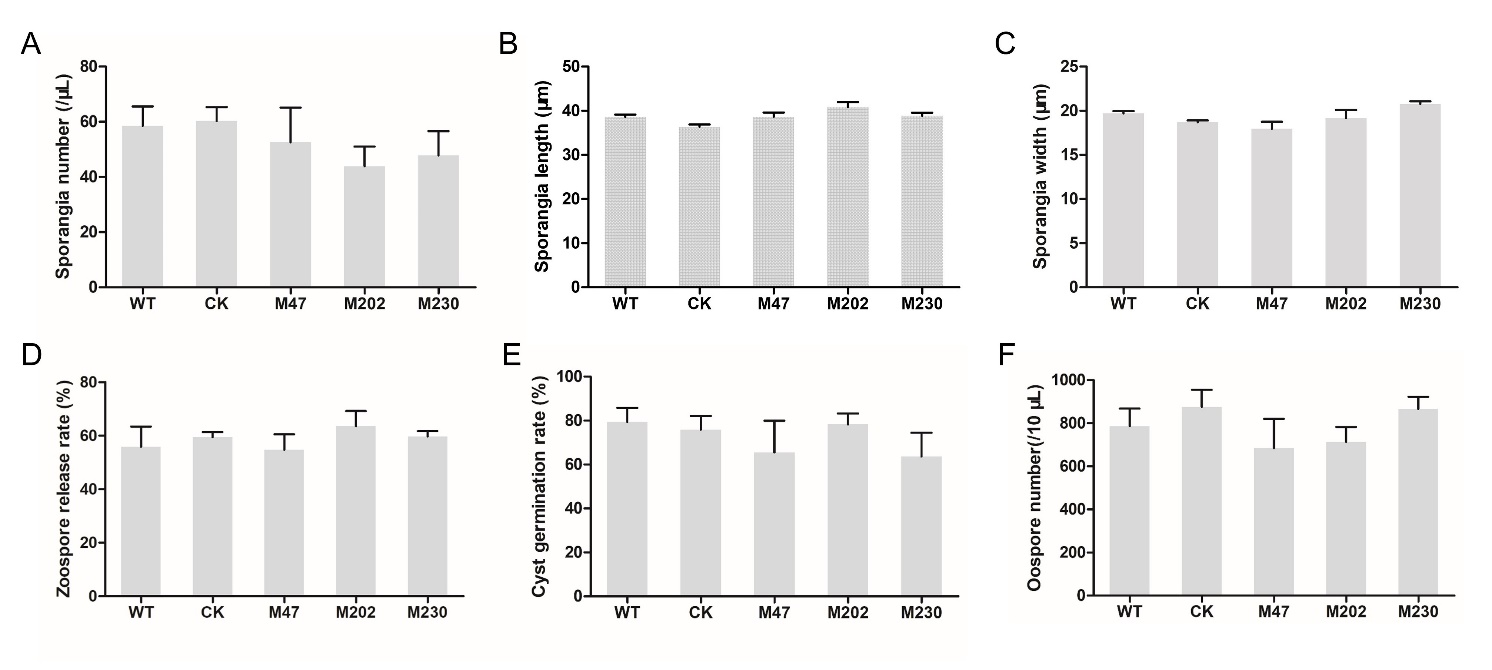


Supplementary Figure S2. *PlCB5L1* did not affect sporangia formation, zoospore release, cyst germination rate and oospores production. (A) Five hyphae plug (9 mm) were flushed with 2 mL sterilized water, then the sporangia numbers were counted. (B) Sporangium length of each strain. (C) Sporangium width of each strain. (D) Mean zoospore release rate. (E) Mean cyst germination rate. (F) The number of oospores in 10 μL oospores homogenate. Data are mean ± SD (n=9). The data were statistically analyzed with Duncan’s Multiple Range Test method, and no significant difference were found..
